# Website Update: A New Graphical User Interface to EMBOSS

**DOI:** 10.1002/cfg.136

**Published:** 2002-02

**Authors:** Tim J. Carver, Lisa J. Mullan

**Affiliations:** MRC UK HGMP Resource Centre Genome Campus Hinxton, Cambridge Cambridgeshire CB10 1SB UK


**Comparative and Functional Genomics**
*Comp Funct Genom* 2002; **3**: 75–78.
**DOI:** 10.1002/cfg.136

**Feature**

### Website Update: A new graphical user interface to EMBOSS

Tim J. Carver* and Lisa J. Mullan
*MRC UK HGMP Resource Centre, Genome Campus, Hinxton, Cambridge, Cambridgeshire, CB10 1SB, UK*

*Correspondence to:
MRC UK HGMP Resource Centre, Genome Campus, Hinxton, Cambridge, Cambridgeshire, CB10 1SB, UK.
E-mail: tcarver@hgmp.mrc.ac.uk*

**Keywords:** EMBOSS; Bioinformatics; HGMP; graphical interface; Java

Received: 1 December 2001
Accepted: 4 December 2001
Published online:
17 January 2002

EMBOSS, the European Molecular Biology Open Software Suite is a collection of over 150 bioinformatics programs. It provides individual applications for retrieval, editing, and analysis of nucleotide and peptide sequences. It also includes software for analysing protein structure. Distributed under the GNU General Public License software agreements [6], all programs are available, free of charge, to all academic or commercial users. EMBOSS brings together programs from various sources including the EMBOSS development team, individuals and members of the EMBnet community.

To mark the fifth anniversary of EMBOSS, an exciting new graphical user interface (GUI), 'Jemboss', has been launched. Written at the HGMP-RC, in collaboration with the core EMBOSS development team, it has been designed to appeal to a broad base of research biologists wishing to utilise the computational analysis applications that EMBOSS offers. Jemboss is a Java application and has been designed to run on UNIX workstations, proprietary PC operating systems or indeed any Java enabled platform. This user-friendly interface dispenses with the requirement for training in UNIX or operating EMBOSS programs via a command line. It may therefore be used by biological scientists with the minimum of computing experience.

A server for the new interface, accessible to registered HGMP users, has been set up at the EMBOSS web site [5]. It is also linked from the 'Bioinformatics Applications' menu on the HGMP site. Jemboss will function in client-server mode allowing many users to run their analyses remotely. The Jemboss interface window (the client) is launched on the users' workstations. Analyses are then run on the HGMP machines and the results returned to the local screen. This has been achieved using the Simple Object Access Protocol (SOAP) [1], a new advance in Internet Communication Technology designed specifically to circumvent firewall restrictions. This, therefore, allows the user access to the EMBOSS applications whilst still maintaining the security of the server computer. SOAP manages this by using HyperText Transfer Protocol (HTTP), which is the protocol used to transfer data on the World Wide Web.

Jemboss is also incorporated into the general EMBOSS distribution, packaged together with all the EMBOSS applications. System administrators who provide a local copy of EMBOSS on their site therefore also have the opportunity of making the GUI available to local users. In this standalone mode it will access and run a local copy of the EMBOSS programs, the programs are called directly and there is no need for the SOAP calls.

Copyright © 2002 John Wiley & Sons, Ltd.

In the client-server mode from the HGMP, Jemboss is launched from the web site using Java Web Start, which must be downloaded onto the user's computer in order to create a Java runtime environment for the Jemboss interface. This type of launch has the advantage of ensuring that the most recent version of EMBOSS is available behind the GUI. The first time Jemboss is launched there may be a delay whilst several files are transferred to the local computer. The user is then presented with a structured list of EMBOSS programs (see Figure 1A,i) at the left of the window. Grouped in accordance with current EMBOSS functional characterisations, programs are listed by name, together with a short description. Below these menus the software applications are listed in alphabetical order (ii). The user may scroll down this menu to select the required program. Alternatively, the name of an application may be typed into the 'Go To' field (iii). A blue marker will highlight various programs as the name is typed into the field. The function you require becomes highlighted as you type the first unambiguous letter of its name. Selection of the program is done using a mouse click. The program form (iv) is displayed in the central Jemboss panel. At the head of the form are the name of the application and a single line description of its function.

A tab in the bottom right hand corner of the program form (v) opens the file manager to display the files and directories on the local disc. Files may be viewed and edited by double clicking on the filename. Remote files are accessed via a remote file manager launched from the pull down 'File' menu on the Jemboss tool bar. Data may be used from either the user's home directory, or the appropriate scratch directory (Figure 1B,vi). Files may be dragged between file managers or into the sequence input field of the program form.

Input sequences are retrieved from user files or database entries and may be tailored to extract a specific region of a desired sequence. The 'Input Sequence Options' window allows users to select a database and/or input format from pull-down menus. If a database has been selected in this window, it will automatically appear in the 'Sequence Filename' field of the program form and will be displayed as the name of the database followed by a colon. The user must then specify, directly after this colon, the accession number, or identifier of the sequence to be retrieved or analysed. Failure to do this will produce an error message. Wildcards are also allowed in this text field, and are represented, as they would be on the command line, by an asterisk (*).

Sequences can be presented to Jemboss in a number of ways such as a database name, sequence filename, or cut and pasted into the sequence entry part of the form. A list of database sequence entries or sequence files can be entered by dragging them

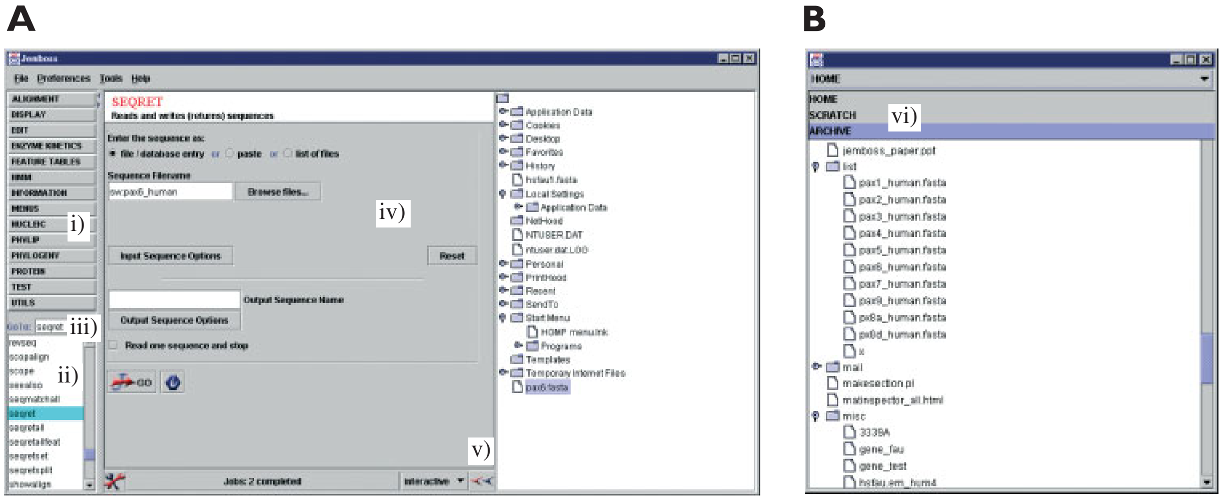

**Figure 1. A** Jemboss - the new graphical user interface for the European Molecular Biology Open Software Suite. **B** Remote file manager. Files in both home and scratch directories are accessible if remote files are stored at the HGMP

Copyright © 2002 John Wiley & Sons, Ltd. 

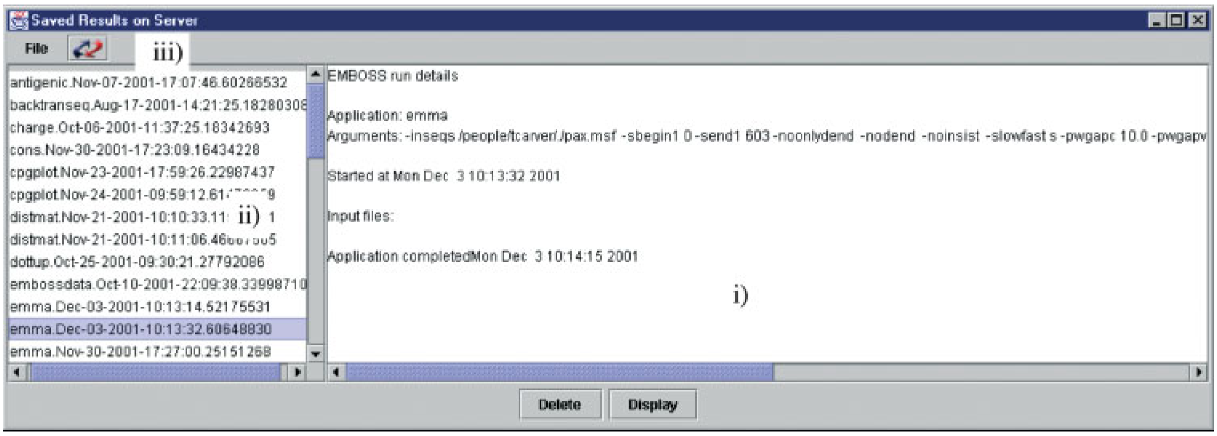

**Figure 2.** The Jemboss project manager. Each application run is stored on the server until deleted by the user into the 'list of files' fields on the program form. Alternatively, a list file containing multiple sequences or database accession numbers can be used. The file can be dragged from either the local or the remote file manager into the sequence input field. These files must be prefixed by an @ sign, in order to identify them as list files to the EMBOSS applications.

EMBOSS is designed to automatically calculate some sequence specific parameters and, in a command line environment, set some defaults or limits on-the-fly. Jemboss emulates this behaviour using the Java Native Interface [4] which accesses the EMBOSS libraries located on the remote server. Redundant parameters for an individual analysis become disabled. Options still available to the users appear as default settings. These may be altered within defined limits.

In this first release of Jemboss, a simple project management system (Figure 2) has been put into place. Each EMBOSS application run is allocated its own working area on the remote server, enabling storage of information specific to each run (i) including input sequences and the parameters used. This storage of data is irrespective of whether an application is run interactively or in the background (as described later). Applications are listed in alphabetical order (ii), with the most recent process at the head of a section. The refresh button (iii) may be used to update this list at any time during a session. Results may also be retrieved at any time, and information on each run is stored until deleted by the user.

A job manager (Figure 3) has also been implemented in this Jemboss release. Located at the bottom of the Jemboss window, it allows the user to run a process interactively or in the background (as a batch job). The progress of all batch runs is monitored by the job manager, which then logs all applications run in a session in a similar manner to the results saved on the server. The status of each run is visible on the job manager bar. Each application within EMBOSS has been set to run in a default mode (i.e. batch or interactive), but this may be altered at the users' discretion.

The results window (Figure 4) for an interactive application appears automatically when the process has completed. Two tabs are present for interactive

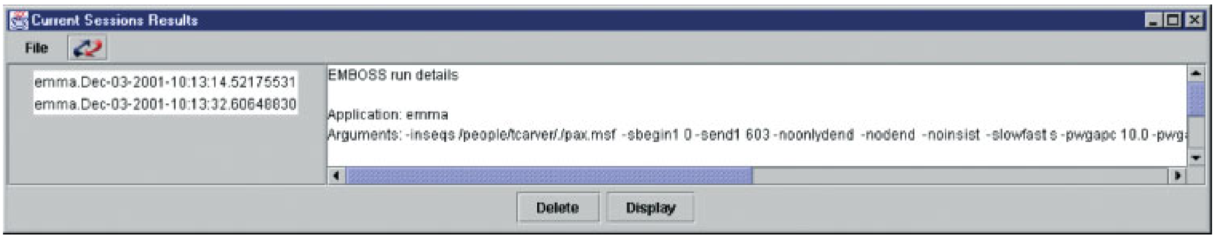

**Figure 3.** The job manager logs all programs run in a single session. Results may be displayed at any time

Copyright © 2002 John Wiley & Sons, Ltd. 

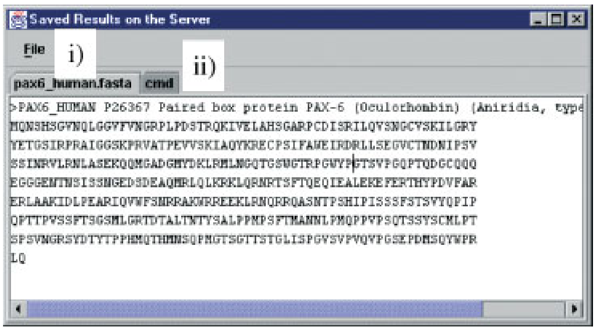

**Figure 4.** The results window displays all input and output information in individual tabbed windows processes to accommodate the results data (i) and EMBOSS command line syntax (ii). Batch job results are accessed via the job manager. These results also include tabs to represent the data in the input and output files.

Java was initially chosen as the programming language for Jemboss to allow the interface to be platform independent. In addition to interfacing with the EMBOSS programs, which are written in C, Jemboss can also be a portal to other bioinformatics tools. The first addition is Jalview [3], which is a multiple sequence alignment viewer and editor, written in Java and is intended to be used in conjunction with the EMBOSS multiple sequence alignment program emma. After aligning a set of sequences, Jalview can be launched using the 'Tools' menu on the Jemboss window. The alignment file is dragged into the Jalview window where the alignment may be viewed and manually edited. Alignments may be saved in postscript format for eventual printing or pasted into a document. Unlike current alignment viewers offered to users of EMBOSS, such as showfeat or prettyplot, Jalview allows users to colour residues according to various physical properties of amino acid residues and calculate a consensus view of all aligned sequences.

Documentation for each program is provided on several levels. Information, such as minimum and maximum parameter values, is provided for each of the variables on the program form and is indicated in blue under the parameter name. Further information on the function of certain parameters may be accessed, using the 'mouse over' facility, by placing the cursor over the name of the parameter. The full EMBOSS documentation, which is also available on the web [2], is accessed by clicking the information button at the bottom of each program form. A separate window will display all the available information on the application.

Jemboss has been designed and implemented in a relatively short time and work is on-going to improve it. The interface is seen as an opportunity for biologists to more easily avail themselves of current computing technology in order to advance their work. Knowledge of UNIX is no longer a prerequisite to use this free software and use of the programs is intended to be as intuitive as possible. If you have any suggestions about Jemboss that you feel would be of benefit to yourselves and other users then please let us know.

### Acknowledgement

The authors would like to thank all the EMBOSS development team for their suggestions in the design of this software. In particular Alan Bleasby for his suggestions in a number of areas and the code he has provided to enable Jemboss to utilise all the sequence file formats EMBOSS supports.

### References

1. Apache Software Foundation: **http://xml.apache.org**
2. EMBOSS documentation: **http://www.uk.embnet.org/Software/EMBOSS**
3. Jalview, a multiple alignment editor: **http://jura.ebi.ac.uk:6543/jalview**
4. Java Native Interface Sun Microsystems Inc.:**http://java.sun.com/j2se/1.3/docs/guide/jni/**
5. Jemboss website: **http://www.uk.embnet.org/Software/EMBOSS/Jemboss**
6. Gnu General Public License: **http://www.gnu.org/licenses/licenses.html**

Copyright © 2002 John Wiley & Sons, Ltd. 

